# Electrophoretic Deposition of Hydroxyapatite Film Containing Re-Doped MoS_2_ Nanoparticles

**DOI:** 10.3390/ijms19030657

**Published:** 2018-02-14

**Authors:** Hila Shalom, Yishay Feldman, Rita Rosentsveig, Iddo Pinkas, Ifat Kaplan-Ashiri, Alexey Moshkovich, Vladislav Perfilyev, Lev Rapoport, Reshef Tenne

**Affiliations:** 1 Department of Materials and Interfaces, Weizmann Institute, Rehovot 76100, Israel; hila.shalom@weizmann.ac.il (H.S.); rita.rosentsveig@weizmann.ac.il (R.R.); 2 Department of Chemical Research Support, Weizmann Institute, Rehovot 76100, Israel; Isai.Feldman@weizmann.ac.il (Y.F.); iddo.pinkas@weizmann.ac.il (I.P.); ifat.kaplan-ashiri@weizmann.ac.il (I.K.-A.); 3 Department of Science, Holon Institute of Technology, P.O. Box 305, 52 Golomb St., Holon 58102, Israel; alexeym@hit.ac.il (A.M.); vladper@hit.ac.il (V.P.); rapoport@hit.ac.il (L.R.)

**Keywords:** hydroxyapatite, electrophoretic deposition, nanoparticles, inorganic fullerene-like, tribology

## Abstract

Films combining hydroxyapatite (HA) with minute amounts (ca. 1 weight %) of (rhenium doped) fullerene-like MoS_2_ (IF) nanoparticles were deposited onto porous titanium substrate through electrophoretic process (EPD). The films were analyzed by scanning electron microscopy (SEM), X-ray diffraction and Raman spectroscopy. The SEM analysis showed relatively uniform coatings of the HA + IF on the titanium substrate. Chemical composition analysis using energy dispersive X-ray spectroscopy (EDS) of the coatings revealed the presence of calcium phosphate minerals like hydroxyapatite, as a majority phase. Tribological tests were undertaken showing that the IF nanoparticles endow the HA film very low friction and wear characteristics. Such films could be of interest for various medical technologies. Means for improving the adhesion of the film to the underlying substrate and its fracture toughness, without compromising its biocompatibility are discussed at the end.

## 1. Introduction

Self-lubricating solid-state films are used for a variety of applications where fluid lubricants can-not support the excessive load or are prohibitive. Two examples for the use of such films are, under vacuum or low temperature conditions, where lubrications by fluids are not relevant. Other uses include the automotive, medical devices, power generation, machining, shipping, aerospace industries as well as many others [1]. Often such films are in fact a nanocomposite made of hard matrix containing a minority phase of a soft metal like copper or silver, or impregnated nanoparticles with good tribological performance [2,3,4,5,6]. More recently, self-lubricating films containing carbon nanotubes [7], MoS_2_ [8] and WS_2_ [9] nanoparticles have been described.

Hydroxyapatite (HA, Ca_10_(PO_4_)_6_(OH)_2_) is used as a bone replacement material in a variety of orthopedic implants and artificial prostheses [10,11]. Given the fact that already 15% of the population is above 65 and increasing, artificial orthopedic implants have become a major health issue. However, this material suffers from high wear and poor fracture toughness, which can be improved by composing it with a toughening phase. To alleviate these problems various methods were conceived including incorporation of nanoparticles (NP) into the HA films. In particular, HA films containing carbon [12] and boron nitride nanotubes [13] were prepared by spark plasma sintering technique. Among the different methods to prepare HA films on metallic substrates, electrophoretic deposition (EPD) is well established and documented in the literature [14,15,16].

Frequently, HA phase also contains associated minerals and materials, including brushite and portlandite. Brushite—(CaH(PO_4_)·2H_2_O) is a metastable compound in physiological conditions and therefore it transforms into hydroxyapatite after implantation of a prostheses [17]. In one report, HA was synthesized in a hydrothermal reaction of CaO and monetite (CaHPO_4_). High concentration of calcium oxide in the reaction led to the formation of excess portlandite—Ca(OH)_2_, while low concentration of calcium oxide resulted in hydroxyapatite [18]. Biphasic calcium phosphate (BCP) is an intimate mixture of two phases of HA and β-TCP (Ca_3_(PO_4_)_2_) in variety of ratios, which appears after annealing of HA above 700 °C [19,20].

**Figure 1:**
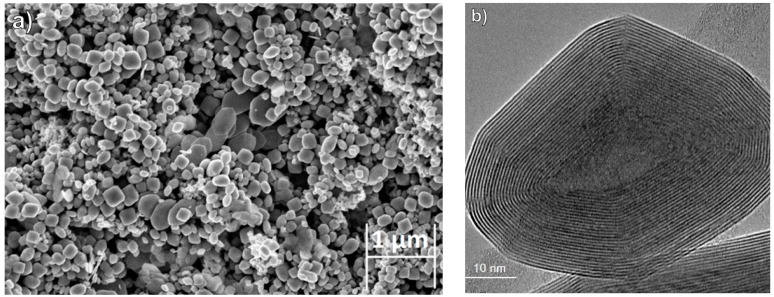
(**a**) High-resolution scanning electron microscopy (HRSEM) image of Re:IF-MoS_2_ nanoparticles powder in In-lens detector 5 kV; (**b**) high-resolution transmission electron microcopy (HRTEM) image of Re:IF-MoS_2_ nanoparticle (see experimental section for details).

Nanoslabs (graphene-like) of MoS_2_ and numerous other layered materials are currently studied intensively for variety of optoelectronic as well as for energy harvesting and energy-storage devices [21]. WS_2_ and MoS_2_ nanoparticles with fullerene-like (IF) structure were also synthesized and were found to perform well as solid lubricants [22,23]. They are presently used in various commercial products, mostly as additives to lubricating fluids, greases, metal working fluids and in high performance bearings (available online at: www.nisusacorp.com and www.utausa.com). Self-lubricating films containing these NP were obtained by co-deposition of the IF NP and various metallic films [24,25,26]. Recently, doping of IF-MoS_2_ nanoparticles with minute amounts (<200 ppm) of rhenium atoms (Re:IF-MoS_2_) was demonstrated [27,28,29,30]. Figure 1a shows high-resolution scanning electron microscope (HRSEM) micrograph of the Re-doped IF NP powder. The oblate shape of the nanoparticles with smooth surfaces is clearly delineated. The size range of the nanoparticles is 70–170 nm with a minor content (<10%) of NP larger than 200 nm. Figure 1b shows high-resolution transmission electron microscopy (HRTEM) image of one such nanoparticle made of some 20 closed and nested layers of MoS_2_. The crystalline perfection and atomically smooth (sulfur-terminated) surface of the IF NP contributes to their excellent mechanical [31] and tribological performance [27,30]. The synthesized nanoparticles are highly agglomerated (see Figure S1) and must be deagglomerated before use. However, in general the agglomerates are weakly bound, and hence only light sonication suffices to disperse them well in aqueous or ethanolic suspensions, which is particularly true for the Re-doped IF-MoS_2_ nanoparticles.

Zeta potential (ZP) analysis, showed that the IF NP have an excess negative charge on their surface [28,29]. The negative surface charge was attributed to the presence of intrinsic defects (sulfur vacancies) as well as the extra charge induced by the ionized doping (Re) atoms. Furthermore, the negative surface charge, particularly in the Re:IF-MoS_2_ NP was shown to induce self-repulsion and formation of stable suspensions of the colloidal nanoparticles in different fluids. Consequently, when added to lubricating fluids and medical gels the Re:IF-MoS_2_ NP produced exceedingly small friction and wear, compared to the undoped nanoparticles and microcrystalline WS_2_ and MoS_2_ [29].

One of the most critical aspects of the usage of nanomaterials is their toxicity and biocompatibility. Several studies deliberated on this issue for MoS_2_ and WS_2_ NP and their IF structure, in particular [32,33,34]. In contrast to various other nanoparticles, the IF NP were found to be non-toxic in general, up to a very high dosage (>100 μg/mL). These encouraging findings could be beneficial for the development of medical technologies based on such nanoparticles [30].

In the present work, HA films impregnated with Re-doped IF-MoS_2_ (Re:IF-MoS_2_) NP were prepared by electrophoretic deposition on a porous TiO_2_ substrate, obtained via anodization in fluoride solution. The films were characterized by a number of techniques and their tribological performance was evaluated. The addition of small amounts of the above nanoparticles to the HA films led to substantial improvement in their tribological behavior. Future research into ameliorating the mechanical properties of the films and their biocompatibility is discussed in brief. Such films could, potentially be useful in the future for orthopedic implants, which in general suffer from poor wear resistance.

## 2. Experimental

### 2.1. Sample Preparation

#### 2.1.1. Surface Treatment

Anodization of titanium surface produces a highly-textured surface comprising an organized array of TiO_2_ nanotubes [35,36,37]. Prior to the anodization, each titanium electrode (30 × 5 × 0.3 mm, 97 wt % purity) was polished with silicon carbide paper to a mirror finish. It was subsequently cleaned by sonicating in a series of solvents, i.e., acetone, ethanol, methanol, isopropanol and finally distilled water, then dried under a nitrogen stream.

#### 2.1.2. Titanium Anodization

An electrochemical cell containing two-electrodes, i.e., platinum (cathode) and titanium (anode) was used. The electrolyte solution contained 1 M (NH_4_)_2_SO_4_ and 0.5 wt % NH_4_F. All electrolytes were prepared from reagent grade chemicals and deionized water. The electrochemical treatment was conducted with a DC power source operated at 2.5 V and 1.5 A, at room temperature for 2.5 h. After the electrochemical treatment, the samples were rinsed with deionized water and dried under nitrogen stream.

#### 2.1.3. Electrophoretic Deposition

The detailed synthesis of the Re:IF-MoS_2_ nanoparticles (Re content < 0.1 at %), which were added to the coating processes, was reported before [38]. Three different chemical baths were used for electrophoretic ‎deposition of HA + IF NP on the porous titanium substrate [39]. Titanium samples were used as the working electrode (cathode), while a platinum plate served as the anode. The final volume of all three electrolyte solutions containing 1 mg of the IF NP was 50 mL.

Solution A: The electrolyte solution consisted of 42 mM Ca(NO_3_)_2_ and 25 mM NH_4_H_2_PO_4_, 1 mg Re:IF-MoS_2_ sonicated in 3 mM cetyl trimethylammonium bromide (CTAB). Ethyl alcohol was added into the above solution in a 1:1 ratio in order to reduce the hydrogen evolution on the titanium electrode [40]. The initial pH of the electrolyte solution was 4.5. The coating process was carried out at 40 °C with a DC power supply at 20 V bias and 0.11 A for 3 h. The samples were washed with deionized water and dried for 24 h at 100 °C.

Solution B: The electrolyte solution consisted of 5.25 mM Ca(NO_3_)_2_, 10.5 mM NH_4_H_2_PO_4_, and 150 mM NaCl. The initial pH of the solution was adjusted to 5.30 by adding NaOH. 1 mg Re:IF-MoS_2_ was sonicated in distilled water for 15 min and added to the electrolyte solution. The coating process was conducted with a DC power source operated at 2.5 V and 0.11 A at room temperature for 3 h.

Solution C: The electrolyte solution consisted of 3 mM Ca(NO_3_)_2_ and 1.8 mM KH_2_PO_4_, 1 mg Re:IF-MoS_2_ sonicated in 3 mM CTAB. The initial pH of the electrolyte solution was 5. The coating process was conducted with a DC power source operated at 6 V and 1 A at room temperature for 1 h. The resulting samples, after coating, were washed with deionized water and dried in room temperature.

The bath showing the most uniform coating and good adhesion (solution A) was then further studied by changing the deposition time to 2, 3 and 4 h and subsequent annealing at 700 °C for 1 h. Obviously this process involved a lot of trial and error, using different parameters for the EPD process and annealing. Ultimately, the optimized coating procedure exhibited also the best tribological performance.

### 2.2. Characterization

#### 2.2.1. High-Resolution Scanning Electron Microscopy (HRSEM) and High-Resolution Transmission Electron Microcopy (HRTEM)

The surface morphology of the titanium samples was analyzed by (HRSEM) (Zeiss Ultra 55) after each step. For topographical information, the secondary electrons were recorded using the SE2 and In-lens detectors. For atomic number contrast the backscattering electron (BSE) detector was used. In order to avoid the sample charging during the analysis, the imaging was done under relatively low accelerating voltage (2–5 kV) and low current. Energy dispersive spectroscopy (EDS) analysis (EDS Bruker XFlash/60mm) of the samples was undertaken as well. The reported results of the EDS were based on standard-less analysis and hence is semi-quantitative in nature.

TEM was performed with a JEOL 2100 microscope (JEOL Ltd., Tokyo, Japan) operating at 200 kV, equipped with a Thermo Fisher EDS analyzer. High-resolution TEM (HRTEM) images were recorded with a Tecnai F30 UT (FEI) microscope (FEI, Eindhoven, the Netherlands) operating a 300 kV. The TEM grids were prepared by dripping an ethanolic solution of the nanoparticles onto a collodion-coated Cu grids.

#### 2.2.2. X-ray Diffraction (XRD)

The film was removed from the Ti substrate and carefully crushed into a powder. The powder was analyzed by X-ray powder diffraction (XRD) using TTRAX III (Rigaku, Tokyo, Japan) theta-theta diffractometer equipped with a rotating copper anode X-ray tube operating at 50 kV/200 mA. A scintillation detector aligned at the diffracted beam was used after a bent Graphite monochromator. The samples were scanned in specular diffraction mode (θ/2θ scans) from 10 to 80 degrees (2θ) with step size of 0.025 degrees and scan rate of 0.5 degree per minute. Phase identification and quantitative analysis were performed using the Jade 2010 software (MDI) (available online: http://ksanalytical.com/jade-2010/) and PDF-4+ (2016) database (available online: http://www.icdd.com/products/pdf4.htm).

#### 2.2.3. Raman Spectroscopy

Raman spectra of the powders ground from the films (see Section 2.2.2) were obtained with Horiba-Jobin Yivon (Lille, France) LabRAM HR Evolution set-up using solid state laser with a wavelength of 532 nm. The instrument was equipped with Olympus objectives MPlan N 100 × NA 0.9. The measurements were conducted using a 600 grooves/mm grating. Each spectrum was acquired for 20 s and the spectra were averaged 100 times, which enabled using low excitation power thereby preserving the sample integrity. The spectral ranges collected were from 100 to 1800 cm^−1^.

#### 2.2.4. Zeta Potential Measurements

The surface charge of the HA suspension with and without the nanoparticles was determined by zeta potential (ZP) measurements using ZetaSizer Nano ZS (Malvern Instruments Inc., Malvern, UK) with a He-Ne light source (632 nm). To prepare the samples for these measurements, IF (0.6 mg) NP were deagglomerated in 20 mL purified water by sonicating for 5–10 min using an ultrasonic bath (see Figure S1 for a typical SEM image of such an agglomerate). Subsequently, 0.2 mL of the IF suspension was added to 1.5 mL aqueous solutions with pH varying from 1 to 12 and sonicated for an extra 5 min. Before the addition of the IF NP, the pH of each solution was adjusted using concentrated NaOH or HCl. The final concentration of the IF NP was 0.004 mg/mL. The ZP of the solutions was measured in a folded capillary cell (DTS1060) made from polycarbonate with gold plated beryllium/copper electrodes.

#### 2.2.5. Tribological Testing

A home-made ball-on-flat rig was used for the tribological tests. The tests were carried-out at room temperature and humidity of ~40%. Each test was repeated 5-times. Tribological tests were performed on the titanium samples at every step of the ‎experimental procedure. The tribological testing was done under dry friction conditions. This testing method utilizes flat lower samples and a ball-shaped upper specimen, which slides against the flat specimen. The two surfaces move relative to each other in a linear, back and forth sliding motion, under a prescribed set of conditions. In this testing method, the load is applied vertically downwards through the ball against the horizontally mounted flat specimen. Two measurements procedures were used in these series of tests. Sliding speed of 0.3 mm/s was common to both series. In one series of measurements the load was 10 g; the diameter of the ball (hard steel—AISI 301) was 10 mm and consequently a Hertzian pressure of 150 MPa was applied on the film (20 cycles). In another series, the load was 20 g, the diameter of the ball 2 mm, i.e., a Hertzian pressure of 600 MPa was applied, and the number of cycles was 100.

## 3. Results and Discussion

### 3.1. SEM Analysis

The surface morphology of the titanium before the pretreatment preceding the anodization is presented in Figure S2a,b in the supporting information—SI. Visibly, the fresh surface was heavily contaminated with a dense network of scratches. After treatment of the titanium (Section 2.1.1), a smooth surface with low density of scratches and clean from contaminants was obtained (Figure S2c,d). The smooth surface was imperative for achieving reproducible tribological measurements.

The surface of the titanium after anodization is displayed in Figure S3. Visibly the anodized titanium surface consists of a dense array of (TiO_2_) nanotubes with the range of pore diameters between 50–130 nm, which form a highly organized, roughly hexagonal, pattern on the Ti surface [35,36,37].

The formal molar Ca/P ratio in HA is 5:3 (1.67). The Ca/P ratio in each coating was calculated based on semi-quantitative EDS analysis. For solution A, the ratio was found to be 2.6. The higher abundance of calcium in this coating could be attributed to the presence of portlandite (Ca(OH)_2_)—see XRD analysis (Section 3.3). The Ca/P ratio of the coating obtained from solution B, which was highly crystalline and discontinuous was 1.5, which agrees well with the HA formula (1.66). The ratio is 1 for the coating obtained from solution C, which can be ascribed to the presence of calcium pyrophosphate phase (Ca_2_(P_2_O_7_)) in the coating—see XRD analysis (Section 3.3).

**Figure 2:**
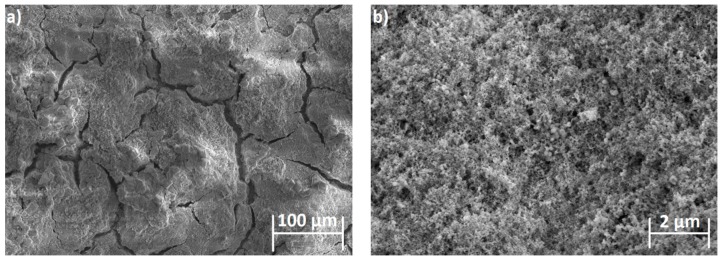
HRSEM pictures of HA with Re:IF-MoS_2_ nanoparticles coating obtained from solution A on porous titanium substrate in two magnifications: (**a**) 100 μm ; (**b**) 2 μm. The film is continuous but visibly is heavily cracked.

It is clear that the surface morphology of the HA film prepared via solutions A (Figure 2) and C was more homogeneous and could be successfully combined with the Re:IF-MoS_2_ NP in the films as opposed to the film obtained from solution B, which was highly crystalline but non-uniform. The surface morphology of the film obtained from solutions B and C are shown in Figures S4 and S5, respectively.

In the next step, the experimental parameters of the Ti-substrate anodization and the electrophoretic deposition from solution A were varied in order to obtain uniform coatings having optimized tribological performance.

**Figure 3:**
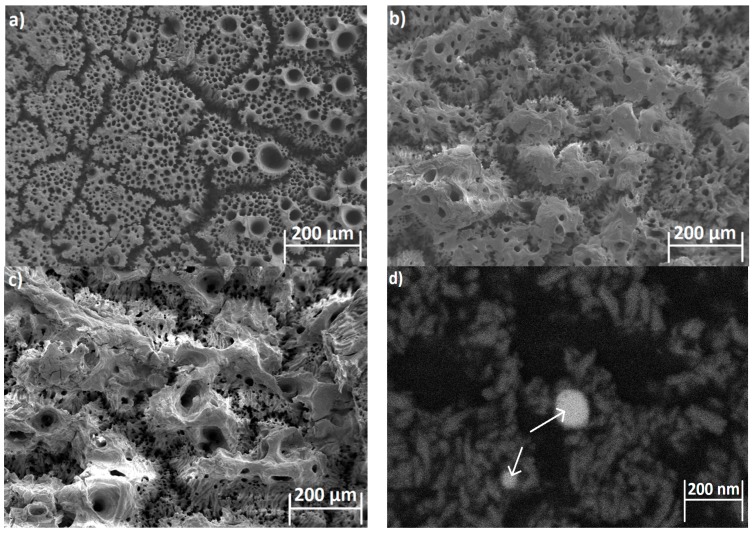
HRSEM images of the HA film with Re:IF-MoS_2_ obtained from solution A after 2 (**a**), 3 (**b**), and 4 h (**c**) deposition. The Re:IF-MoS_2_ nanoparticles in the film (**c**) are observed in the backscattering electron (BSE) mode (**d**). The arrows in Figure 3d point on the Re:IF-MoS_2_ nanoparticles occluded in the HA film.

The SEM images of the surface of the HA films with Re:IF-MoS_2_ nanoparticles obtained from solution A for different deposition periods are shown in Figure 3. The surface of the coated film shows defects, including the presence of cracks and pores with circular shape. Such pores can be probably attributed to the formation of H_2_(g) bubbles during the coating process.

Interestingly, the bias applied during EPD for solution B (and C) was appreciably smaller (2.5 V) compared to solution A (20 V). On the other hand, the film obtained by EPD from solution A was quasi-continuous. It was highly crystalline but less uniform in the case of solution B, i.e., the apparent current density was higher than that calculated on the basis of the formal electrode surface. The higher voltage used for the EPD from solution A implied a much higher rate of hydrogen production, which could explain the porous structure of this film. The density of the pores and their sizes could be possibly tuned by the bias applied on the cathode during the electrophoretic deposition. Furthermore, addition of surface active agents, like CTAB and others, could reduce the size of the pores. This optimization process is reserved for a future study. In any event, the large cracks are diminished, and the pore-size decreased as the coating time was prolonged. The Re:IF-MoS_2_ NP cannot be easily discerned from the HA, due possibly to charging of the film during SEM analysis. Furthermore, the thickness of the coating was a few microns, therefore the nanoparticles could have been buried under the film surface and even be closer to the titanium substrate. Using low energy beam (2 keV) in the BSE mode, the IF NP could be nevertheless observed—see Figure 3d.

### 3.2. Zeta Potential Measurements

**Figure 4:**
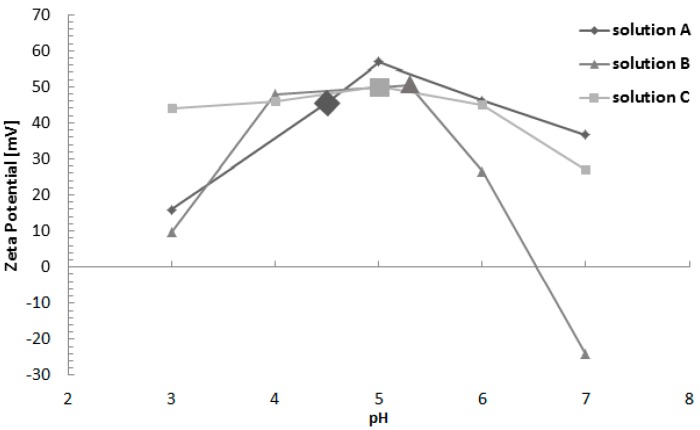
Zeta-potential vs. pH for Re:IF-MoS_2_ nanoparticles. The (positive) ZP of the genuine solutions used for EPD of the HA + IF film are marked by enlarged symbols.

Figure 4 shows the results of the Zeta potential (ZP) measurements performed with the three solutions containing Re:IF-MoS_2_ nanoparticles as a function of pH—up to pH7. The ZP of all the solutions containing the nanoparticles is positive for pH below 6.5. Beyond that pH—the ZP of solution B becomes negative, while that of solutions A and C remain positive. This difference can be attributed to the addition of the CTAB, which is a cationic surfactant, to solutions A and C. The (positive) ZP of the natural solutions used for EPD is marked on Figure 4 for all three solutions.

The ZP measurements show that the species in the HA solution containing the IF NP are positively charged and consequently, the HA film could be deposited on the negative electrode (Ti) during the EPD process. The ZP of the IF NP in pure water, ethanol solution, CTAB in water, and the three solutions used for the EPD (included also in Figure 4) are summarized in Figure S6 of the supporting information, the errors of the ZP measurements is about 2%.

### 3.3. X-ray Diffraction (XRD)

**Figure 5:**
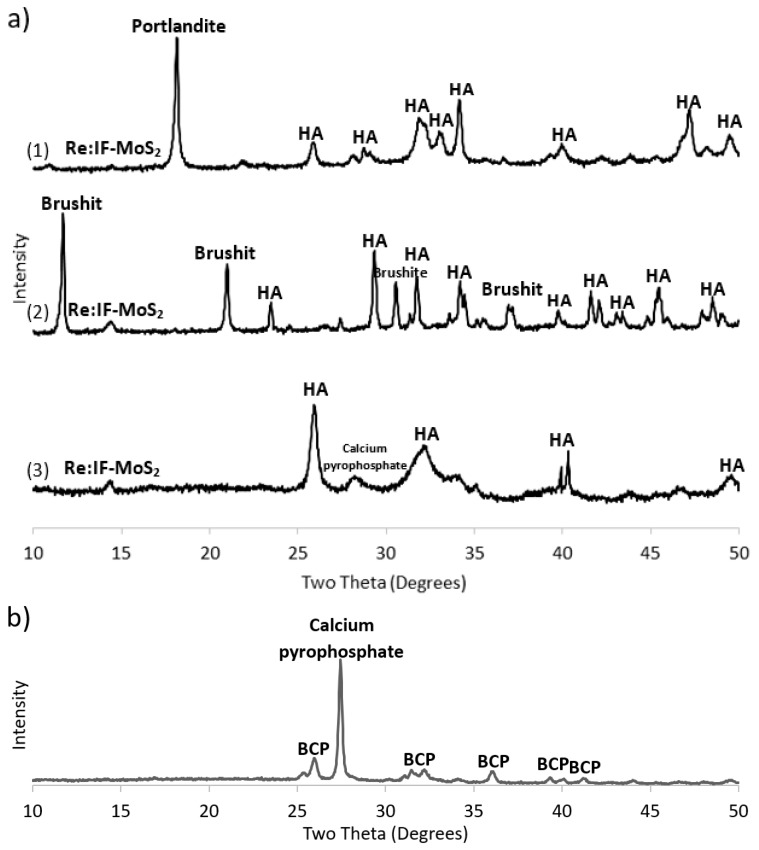
(**a**) XRD patterns of the HA films incorporating Re:IF-MoS_2_ nanoparticles: film obtained from solution A (1), solution B (2) and solution C (3); (**b**) shows the XRD pattern of the film obtained from solution A (3 h) after annealing (700 °C for 1 h). Here, a strong crystalline peak associated with calcium pyrophosphate phase (Ca_2_(P_2_O_7_)) is observed. This phase is obtained through water evaporation from the HA (Ca_10_(PO_4_)_6_(OH)_2_) film. The presence of the Re:IF-MoS_2_ nanoparticles did not change appreciably upon annealing, suggesting that these NP are thermally stable at the annealing conditions.

The results of the XRD analyses are summarized in Figure 5 and in Table 1. The XRD patterns of the different coatings obtained from solutions A, B and C are shown in Figure 5a. The major phase obtained by EPD of these solutions is HA. Nonetheless, the coating obtained from solution A contained appreciable amounts (25 wt %) of portlandite (Ca(OH)_2_). Solution B, on the other hand, contained, in addition to the HA, also significant amounts of brushite—(CaH(PO_4_)·2H_2_O). The film obtained from solution C contained calcium pyrophosphate—(Ca_2_(P_2_O_7_)). The presence of the Re:IF-MoS_2_ nanoparticles in the coatings is confirmed by the tiny peak at 14.3°. The content of the IF NP is calculated as 0.2 wt % for solutions A, 1.5 wt % for solution B and 1.4 wt % for solution C. This amount is rather small but could nevertheless lead to major improvements of the tribological properties of the film without compromising its mechanical robustness.

Following the annealing of the film obtained from solution A (Figure 5b), the HA became biphasic calcium phosphate (BCP) [19], i.e., intimate mixture of two phases: HA (73.6 wt %) and β-TCP (5.9 wt %), and 0.1 wt % Re:IF-MoS_2_ NP.

**Figure 6:**
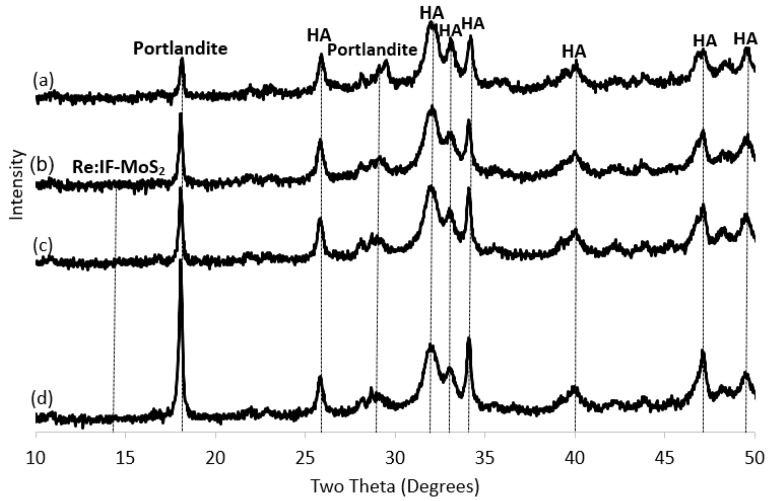
XRD patterns: Films obtained from solution A without the Re:IF-MoS_2_ NP (**a**) and (with the IF NP) for different deposition periods: after 2 h (**b**), 3 h (**c**) and 4 h (**d**).

**Figure 7:**
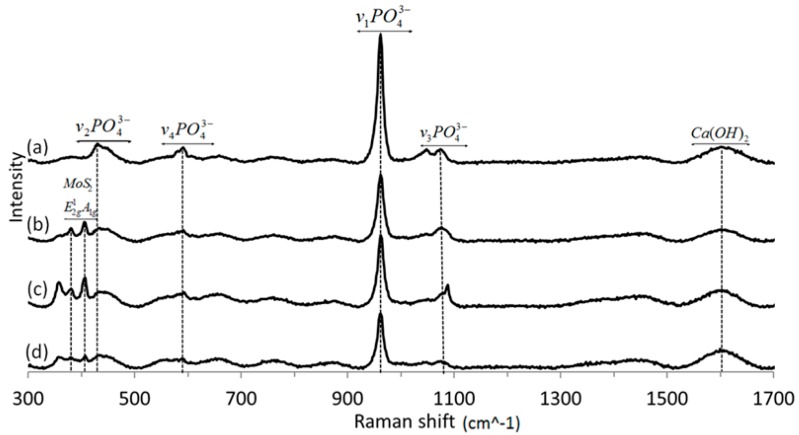
Raman spectra of HA powder film without (**a**) and with the Re:IF-MoS_2_ nanoparticles obtained from solution A for different EPD periods: after 2 (**b**), 3 (**c**) and 4 h (**d**).

The XRD patterns of the films obtained from solution A without the NP (a) and with the IF NP for different deposition times (b–c) is shown in Figure 6. The percentages of the compounds in each film is presented in Table 2. The major phase in the films is hydroxyapatite. However, one can conclude from Figure 6 that the relative amount of the portlandite in the film increases with extending deposition times. The relative amount of the calcium oxide does not seem to vary with the deposition time which is also true for the relative content of the IF NP. Although the signal of the IF NP is non-visible in Figure 6, their presence is confirmed through both electron microscopy (Figure 3) and the Raman measurements (see below Figure 7).

### 3.4. Raman Spectroscopy

The Raman spectra of HA + IF films prepared from solution A for different deposition times (2, 3 and 4 h) are shown in Figure 7. The spectra show the characteristic vibration bands of calcium hydroxide (wide peak at 1600 cm^−1^) and poorly crystalline phosphoric moieties, especially phosphate PO_4_^−3^ bands at 469 (ν_2_), 562–603 (ν_4_), 962 (ν_1_) and 1000–1104 cm^−1^ (ν_3_) [39,41]. These bands are typical of HA. The Raman spectra showed also the typical MoS_2_ modes at 383 (E_2g_) and 408 cm^−1^ (A_1g_) [42,43]. Interestingly, in contrast to the XRD pattern (Figure 6), the Raman bands of the IF NP in the HA film are easily discerned here.

### 3.5. Tribological Testing

Table 3 summarizes the data for the friction coefficient and surface roughness of the different samples under dry conditions. In general, the friction coefficient was found to go down along with the stages of the experimental procedure of preparing the film. The low friction coefficient of the HA film obtained from solution A can be attributed to the IF nanoparticle structure. The nanoparticles exhibit facile rolling when released from the film surface. In addition, gradual peeling/crushing of the NP and material transfer from the film surface to the counter surface of the ball contributed to the facile shearing of the mating surfaces and low friction coefficients. Interestingly, the friction coefficient of the HA film obtained from solution A was maintained also after 700 °C annealing.

Table 4 shows the dry friction coefficient of the coatings obtained from solutions A without (3 h) and with the NP after 2, 3 and 4 h of deposition time on the anodized titanium substrate. Note that, in this series of measurements a higher Hertzian pressure (600 MPa) was used for the tribological test. The dry friction coefficient was reduced with increasing coating-time of the film.

It should be noted that, relative to the previous measurement (see Table 3), there was an increase in the friction coefficient for the HA coating with Re:IF-MoS_2_ nanoparticles obtained from solution A for 3 h deposition time. This result can be accounted for by the weaker adhesion of the coating to the titanium samples under the high pressure (600 MPa) applied onto the film. Nonetheless, following the 4 h deposition time the friction coefficient was very low (0.12) attesting to the quality of the composite film.

Therefore, it is clear that the extended deposition of the composite film resulted in lower friction under very high load. However, the mechanical stability of the film might have been partially compromised. The surface roughness of the films was in the sub-micron range for all the films containing the NP.

**Figure 8:**
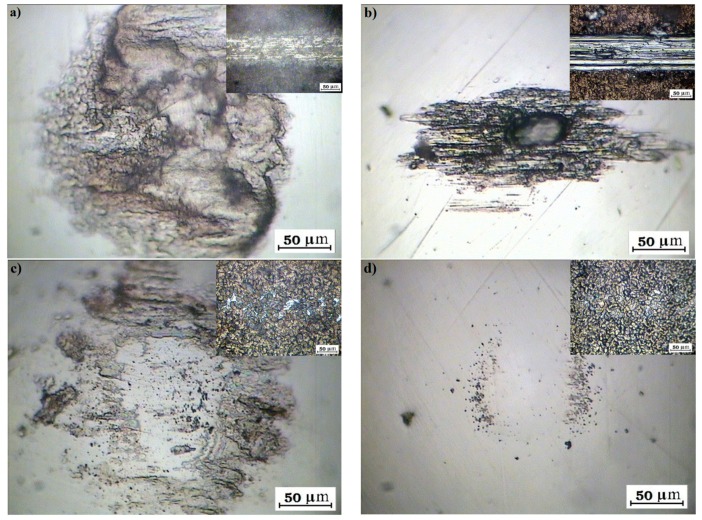
Optical image of wear on the ball and inside the track of HA film without (**a**) and with the Re:IF-MoS_2_ nanoparticles obtained from solution A for different periods: after 2 h (**b**), 3 h (**c**) and 4 h (**d**) on anodized titanium.

Figure 8 shows optical micrographs of the wear of the ball and the wear track on the film (inset) after different periods of EPD (600 MPa) and 100 cycles. In analogy to the friction coefficient, the visible wear scar on the ball and the wear track on the film were markedly reduced with the deposition time of the HA + IF NP film.

## 4. Conclusions

Given the fact that the aging population of the world is approaching one billion people, inabilities due to orthopedic failures and consequently artificial bone implants became a major health concern. Hydroxyapatite (a form of calcium phosphate) is the main constituency of the bone. Therefore, calcium-phosphate coatings occupy important part of modern research in medical technology. Calcium phosphate coatings containing up to 1.5 wt % Re:IF-MoS_2_ nanoparticles were deposited on porous titanium substrate by electrophoretic deposition using DC bias. Three different solutions were used for the deposition. Solution A is based on ethanol-water mixture as solvent, solution B contained sodium chloride and solution C without (ethanol or NaCl) additive. The major phase in each coating was hydroxyapatite which successfully incorporated small amounts of Re:IF-MoS_2_ nanoparticles. The electrophoretic deposition from solution B was found to be highly crystalline and discontinuous, i.e., it did not fully cover the porous titanium substrate. The film obtained from solution C was found to have high friction coefficient. The tribology test performed on the coatings showed lower friction coefficient and wear as the time of the deposition increased beyond 3 h period. The low friction coefficient was maintained ‎also after annealing of the sample (solution A). The good tribological performance of the film indicates also that the film is robust and suffers no adhesion problems during the tests in dry conditions.

The present films must be improved in order to increase the adhesion of the film to the underlying substrate and its fracture toughness, while maintaining its biocompatibility, especially under wet conditions [44]. Future studies will attempt to use a third component, possibly in the form of a biocompatible polymer, which could be suitable as a binder in this case. Remarkably though, the present composite films show very low friction and reasonable adhesion to the underlying rough substrate even under very high load (600 MPa).

## Figures and Tables

**Table 1:** Composition of the films deposited from different solutions determined from the XRD analysis.

EPD Films	HA	Portlandite	Brushite	Calcium Pyrophosphate	β-TCP	Re:IF-MoS_2_
Film obtained from solution A	74.8 wt %	25 wt %				0.2 wt %
Film obtained from solution B	17.2 wt %		81.3 wt %			1.5 wt %
Film obtained from solution C	81.1 wt %			17.5 wt %		1.4 wt %
Film obtained from solution A after annealing	73.6 wt %			20.4 wt %	5.9 wt %	0.1 wt %

**Table 2:** Composition of the film determined via XRD analysis for different deposition times (from solution A).

EPD films	HA	Portlandite	Calcium Oxide	Re:IF-MoS_2_
Film obtained from solution A without Re:IF-MoS_2_ (3 h)	87.8 wt %	4.6 wt %	7.6 wt %	
Film obtained from solution A (2 h)	82.6 wt %	7.4 wt %	9.1 wt %	0.3 wt %
Film obtained from solution A (3 h)	80.4 wt %	11.3 wt %	8.0 wt %	0.3 wt %
Film obtained from solution A (4 h)	77.8 wt %	13.6 wt %	8.3 wt %	0.3 wt %

**Table 3:** Summary of the initial and final friction coefficients and the initial roughness for different stages of preparation of the composite HA + IF film. Measurement conditions: diameter of the test ball 10 mm; load = 10 g (Hertzian pressure—P = 150 MPa).

Tested Film	Initial Coefficient of Friction	Final Coefficient of Friction (after 20 Cycles)	Initial Roughness (μm)
Titanium after surface treatment	0.50 ± 0.01	0. 60 ± 0.02	0.23 ± 0.03
Titanium after anodization	0.15 ± 0.01	0.23 ± 0.03	0.50 ± 0.05
Film of HA with Re:IF-MoS_2_ NP obtained from solution A on anodized titanium	0.11 ± 0.01	0.13 ± 0.01	0.45 ± 0.4
Film of HA with Re:IF-MoS_2_ NP obtained from solution B on anodized titanium	0.21 ± 0.02	0.43 ± 0.08	0.37 ± 0.03
Film of HA with Re:IF-MoS_2_ NP obtained from solution C on anodized titanium	0.37 ± 0.23	0.30 ± 0.18	0.52 ± 0.02
Film of HA with Re:IF-MoS_2_ NP obtained from solution A on anodized titanium after annealing	0.12 ± 0.01	0.11 ± 0.02	0.49 ± 0.7

**Table 4:** The initial and final friction coefficients and the initial roughness of the coating on titanium substrate obtained from solution A for different periods of deposition. Measurement conditions: diameter of the test ball 2 mm; load 20 g and Hertzian pressure of P = 600 MPa.

Tested Film	Initial Coefficient of Friction	Final Coefficient of Friction (after 100 Cycles)	Initial Roughness (μm)
Pure HA film obtained from solution A without NP after 3 h deposition	0.66 ± 0.08	0.78 ± 0.04	1.59 ± 0.28
HA film with Re:IF-MoS_2_ NP obtained from solution A after 2 h	0.75 ± 0.05	0.63 ± 0.03	0.49 ± 0.05
HA film with Re:IF-MoS_2_ NP obtained from solution A after 3 h	0.53 ± 0.03	0.55 ± 0.04	0.57 ± 0.17
HA film with Re:IF-MoS_2_ NP obtained from solution A after 4 h	0.13 ± 0.01	0.12 ± 0.02	0.48 ± 0.02

## Supplementary Materials

Supplementary materials can be found at http://www.mdpi.com/1422-0067/19/3/657/s1.
