# Supplementary material for: Electrophoretic Deposition of Hydroxyapatite Film Containing Re-Doped MoS2 Nanoparticles
Source: Int J Mol Sci. 2018 Feb 14;19(3):657. doi: 10.3390/ijms19030657 (PMC5877518; doi:10.3390/ijms19030657)
Supplement: Supplementary file 1 [file ijms-19-00657-s001.pdf]

International Journal of *Molecular Sciences*

MDPI

Supporting Information

Electrophoretic deposition of hydroxyapatite film containing Re-doped MoS2 nanoparticles

Hila Shalom, Yishay Feldman, Rita Rosentsveig, Iddo Pinkas, Ifat Kaplan-Ashiri, Alexey Moshkovich, Vladislav Perfilyev, Lev Rapoport and Reshef Tenne

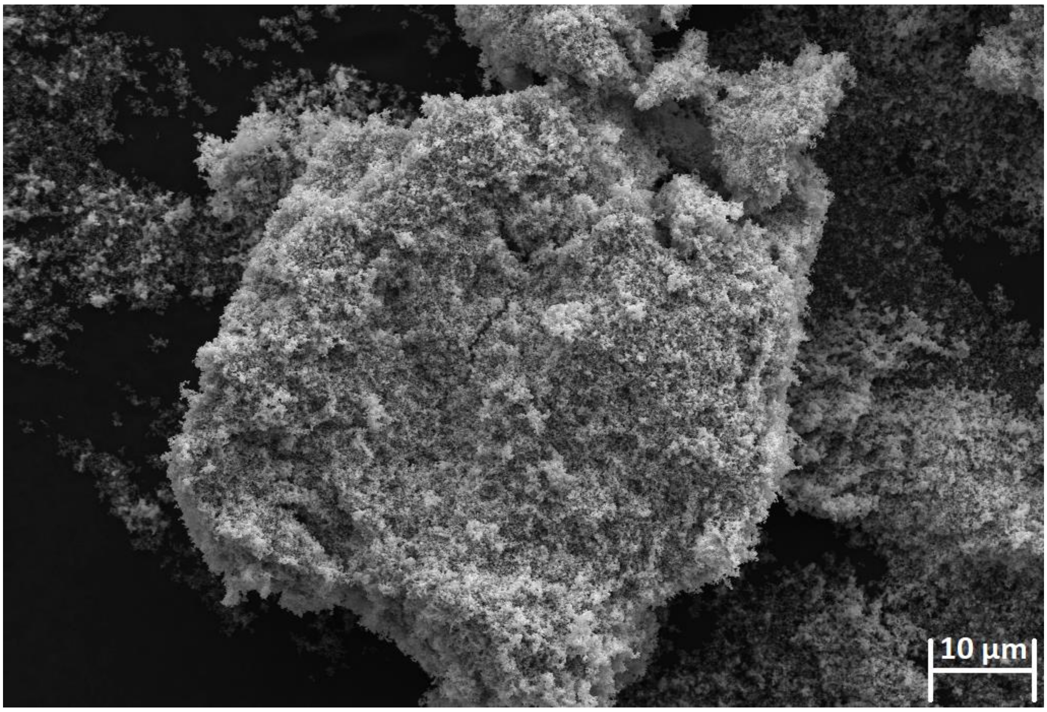

Figure S1. Typical SEM view of an agglomerate of Re:IF-MoS2 nanoparticles.

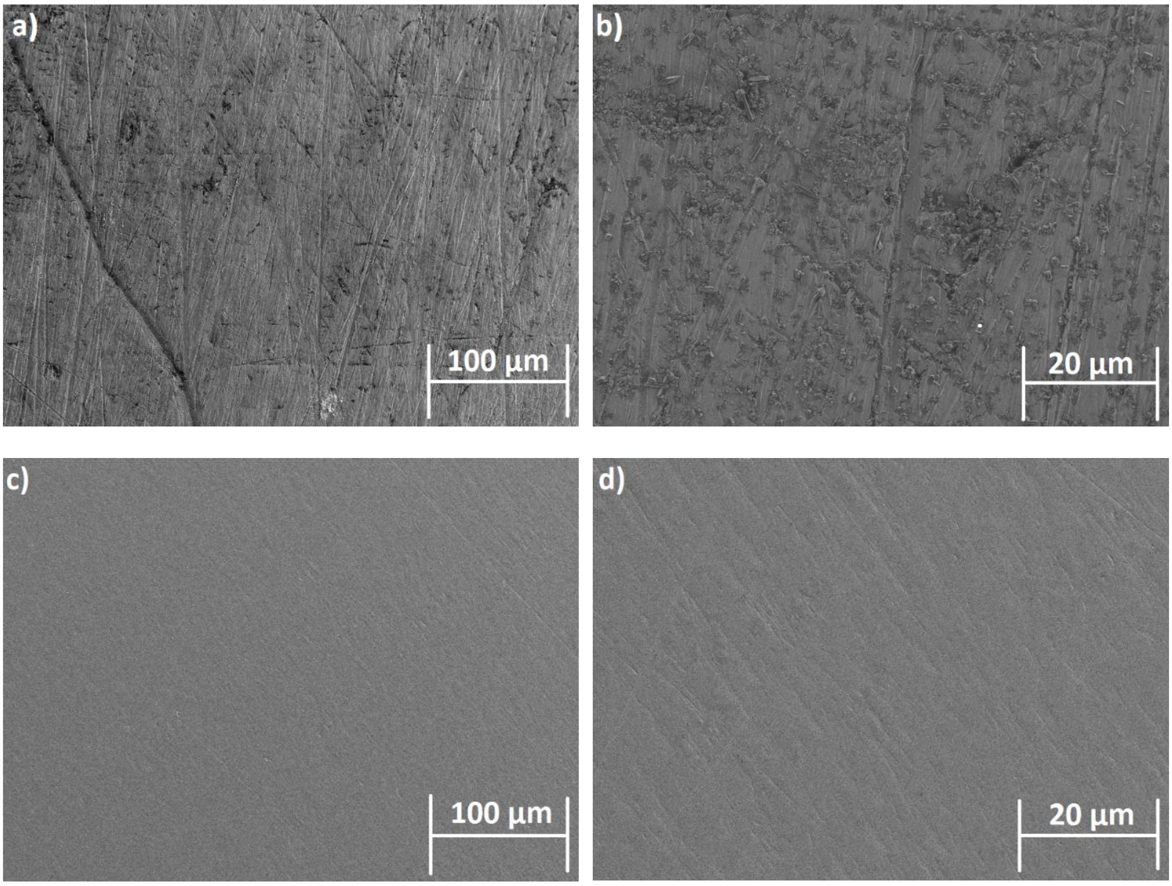

Figure S2. SEM images of titanium surface (**a**,**b**) before and (**c**,**d**) after surface treatment in different magnifications.

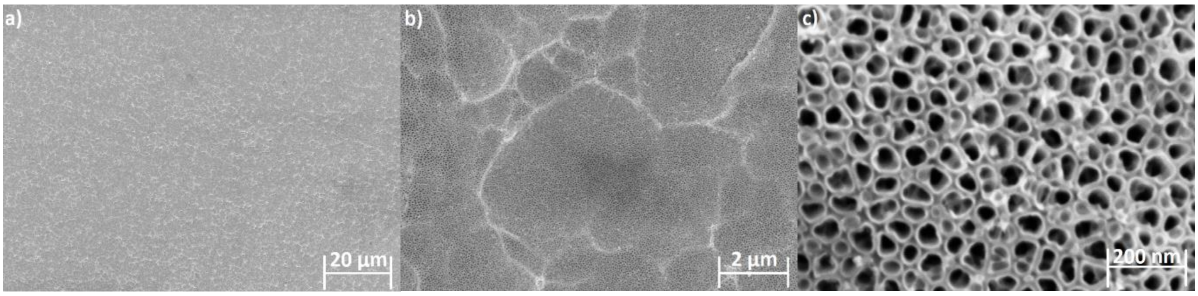

Figure S3. SEM images of porous titanium after anodization in different magnifications, the average diameter of the pores (tubes) is 100 nm.

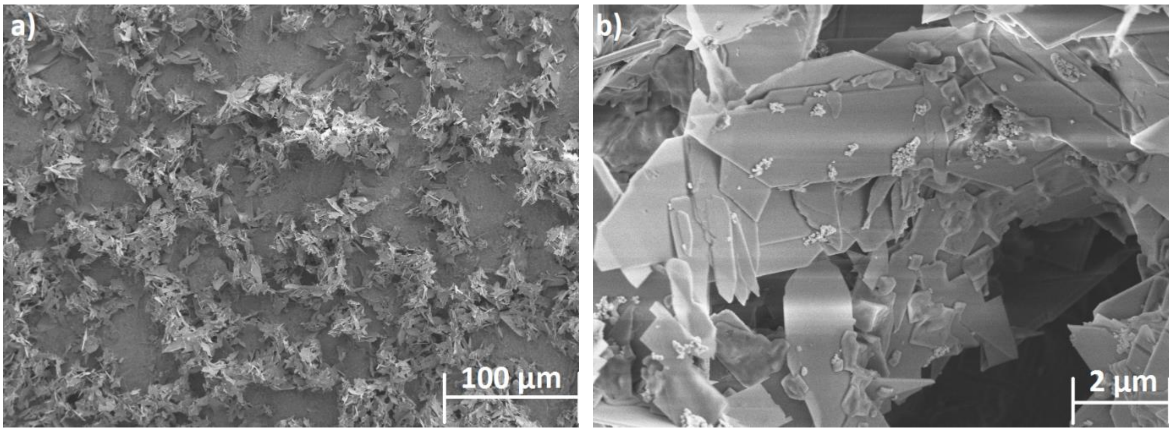

Figure S4. SEM images of HA with Re:IF-MoS_2 nanoparticles coating obtained from solution B (3 h deposition time) on porous titanium substrate in different magnifications.

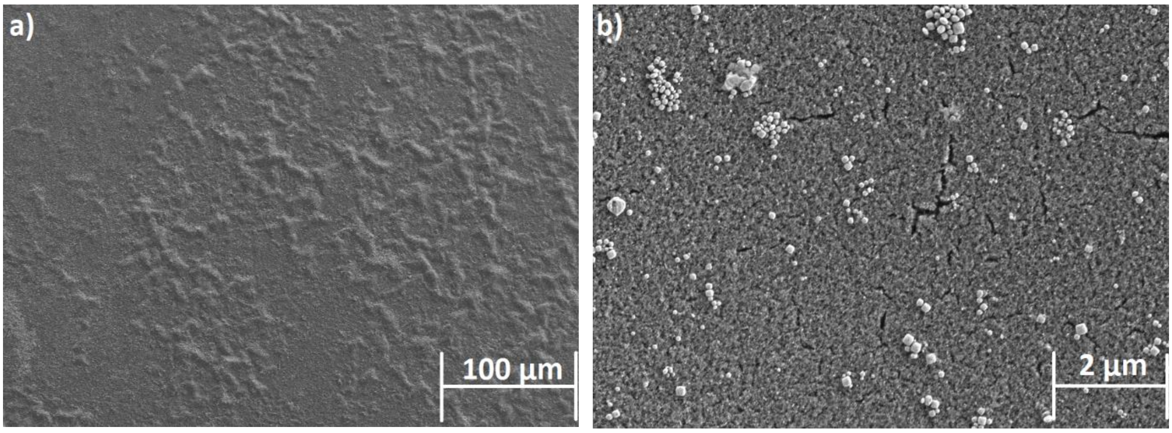

Figure S5. SEM pictures of HA with Re:IF-MoS_2 nanoparticles coating obtained from solution C (1 h deposition time) on porous titanium substrate in different magnifications.

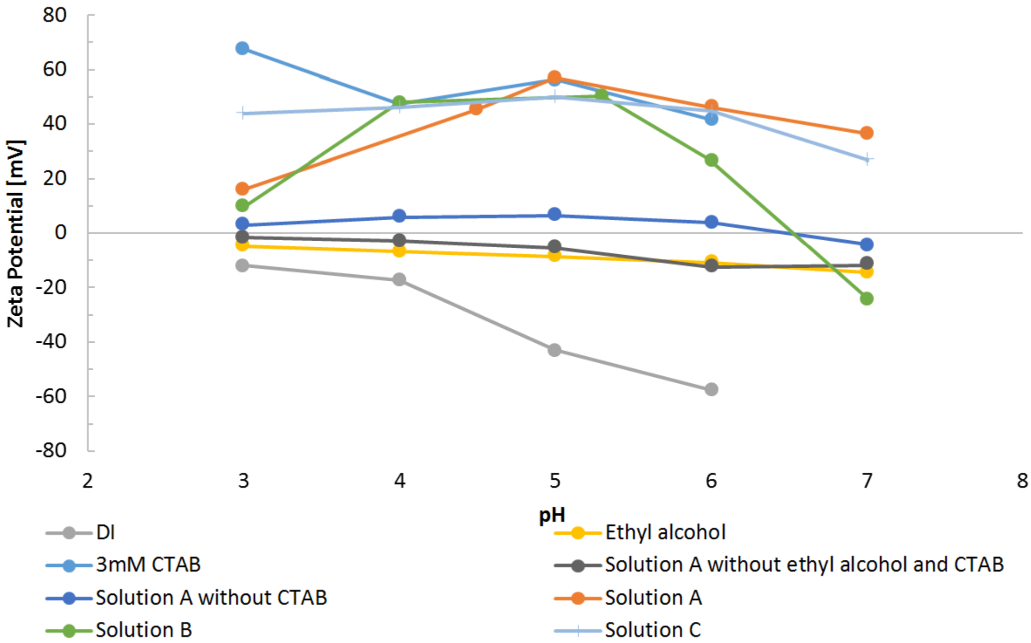

Figure S6. Zeta-potential vs pH for Re:IF-MoS_2 nanoparticles in different solutions.
